# High Dose of Pegylated Interferon for the Treatment of Chronic Hepatitis B in Children Infected With Genotype C

**DOI:** 10.1097/PG9.0000000000000005

**Published:** 2020-08-19

**Authors:** Haruki Komatsu, Ayano Inui, Sachiyo Yoshio, Tatsuya Kanto, Shuichiro Umetsu, Tomoyuki Tsunoda, Tomoo Fujisawa

**Affiliations:** From the * Department of Pediatrics, Sakura Medical Center, Toho University, Chiba, Japan; † Department of Pediatric Hepatology and Gastroenterology, Eastern Yokohama Hospital, Kanagawa, Japan; ‡ Liver Diseases, Research Center for Hepatitis and Immunology, National Center for Global Health and Medicine, Chiba, Japan.

**Keywords:** hepatitis B virus, children, PEG-IFN, high dose, genotype C

## Abstract

Chronic hepatitis B virus (HBV) genotype C infection is unlikely to show a good response to interferon (IFN). However, it is unknown whether a high dose of pegylated IFN (PEG-IFN) treatment would be effective for hepatitis B e antigen (HBeAg)-positive children with chronic HBV genotype C infection.

**Methods:**

HBeAg-positive children and adolescents with chronic HBV genotype C infection were eligible for this study. To increase the dose of PEG-IFN, all patients received PEG-IFN-α-2a (180 μg) without dose adjustment on the basis of body surface area for 48 weeks and were followed up for 24 weeks after the completion of treatment.

**Results:**

Thirteen patients (median age, 9 years) were enrolled prospectively for this study. One patient dropped out, and the remaining 12 patients were evaluated. Of the 12 patients, 11 received PEG-IFN of 180 μg/1.73 m^2^ or more (median, 287 μg/1.73 m^2^). Eight (67%) experienced HBeAg seroconversion, and 1 (8%) achieved hepatitis B surface antigen (HBsAg) loss at the end of follow-up. There was a significant difference in the decrease of hepatitis B surface antigen levels from the baseline to week 24 of treatment between the responders and the nonresponders. Serum cytokines and chemokines were measured in 10 patients. The levels of C-X-C motif chemokine ligand 9, 10, 11, and 13 in the responders tended to be higher than those in the nonresponders during the first 24 weeks of treatment.

**Conclusions:**

A high dose of PEG-IFN treatment was effective and safe. A decrease in the hepatitis B surface antigen level from baseline to week 24 of treatment might be a predictor of HBeAg seroconversion.

## Abstract 2

Supplemental Digital Content is available in the text.

**What Is Known**

- Pegylated interferon (PEG-IFN) treatment is effective for chronic hepatitis B virus (HBV) infection.
- Compared to patients infected with HBV genotype A and B, patients infected with HBV genotype C are unlikely to attain good treatment response to PEG-IFN.

**What Is New**

- A high dose of PEG-IFN treatment for 48 weeks was effective for hepatitis B e antigen (HBeAg)-positive children chronically infected with HBV genotype C.
- A decrease in the hepatitis B surface antigen level from baseline to week 24 of treatment might be a predictor of HBeAg seroconversion in children infected with HBV genotype C.

The major clinical guidelines recommend antiviral treatment for children with chronic hepatitis B virus (HBV) infection who meet the treatment criteria (1–4). Conventional interferon (IFN), pegylated interferon (PEG-IFN), and nucleos(t)ide analogues are available as antiviral treatments for chronic HBV infection in children (1–5). Because of the finite treatment course, durable off-treatment response, and absence of a risk of drug resistance, PEG-IFN is recommended as the preferred initial therapy for children without decompensated liver cirrhosis (1,2). HBV genotype C is the predominant form of HBV in South East Asia (6). However, compared to patients infected with genotypes A and B, patients infected with HBV genotype C are unlikely to attain a good response (7,8). Usually, the dose of PEG-IFN for children is adjusted by body weight (3 μg/kg, maximum 180 μg) or body surface area (BSA) (dose = 180 μg×BSA/1.73 m^2^). Previously, we reported that a high dose of PEG-IFN-α-2a treatment was effective and safe in children with chronic hepatitis C (9). However, it remains unknown whether treatment with a high dose of PEG-IFN could benefit hepatitis B e antigen (HBeAg)-positive children with chronic HBV infection.

We reported that the elevation of C-X-C motif chemokine ligand (CXCL) 9, CXCL10, CXCL11, CXCL13, and interleukin (IL)-21 might be a signature pattern in attaining a functional HBV cure in acute hepatitis B patients and in the chronic hepatitis B patients who were treated with a sequential combination of nucleotide analogues followed by PEG-IFN-α (10). The aim of this study was to determine whether a high dose of PEG-IFN-α-2a treatment would benefit HBeAg-positive children and adolescents chronically infected with genotype C. We also evaluated the dynamics of the serum chemokines associated with HBeAg or hepatitis B surface antigen (HBsAg)seroconversion.

## METHODS

### Study Design

This is a prospective study performed at a single center. Recruitment began in January 2012, and the study ended in December 2019. This study was approved by the ethical committees of Eastern Yokohama Hospital (approval nos. 2016078, 20190036). Written informed consent was obtained from the parents or legal guardians of all patients before they joined the study. Without dose adjustment on the basis of the BSA (180 μg/1.73 m^2^) (5), 180 μg of PEG-IFN-α-2a (Pegasys; Roche, Basel, Switzerland) was administered for children and adolescents once weekly for 48 weeks by subcutaneous injection. PEG-IFN dose reduction was required for a leukocyte count ≤1500/μL, a granulocyte count <750/μL, or a platelet count <70,000/μL. Discontinuation of PEG-IFN treatment was required for a granulocyte count <500/μL or a platelet count <50,000/μL. All patients were followed up for 24 weeks after the completion of 48 weeks of treatment (Supplemental Fig. 1, Supplemental Digital Content, *http://links.lww.com/PG9/A0*). There were no criteria for on-treatment termination rules in this study.

### Patients

Children and adolescents 3 to <20 years of age were eligible if they had been positive for HBsAg for at least 6 months, were positive for HBeAg on 2 occasions within 6 months before enrollment in this study, had a serum HBV DNA level of more than 5 log copies/mL, had 2 episodes of raised serum alanine aminotransferase (ALT) levels (>1 × upper limit of normal [ULN]: male; >43 IU/L, female; >34 IU/L, but ≤10 × ULN) within 6 months before enrollment in this study, and had had findings on a liver biopsy within the previous 6 months that were consistent with the presence of chronic hepatitis B. The reasons for exclusion were co-infection with hepatitis C virus, hepatitis D virus, or human immunodeficiency virus, liver cirrhosis (METAVIR F4 or equivalent) (11), decompensated liver disease, co-existing inherited causes of liver disease, thyroid disease, or cardiac disease, a history of psychiatric disorders and malignant disease, inadequate leukocyte count (≤3000/μL), granulocyte count (≤1500/μL), or platelet count ≤100,000/μL, and suspicion of HCC.

### Evaluation of Treatment Efficacy

The analyses of efficacy included all patients who could be followed for 24 weeks after the completion of the 48-week PEG-IFN treatment. Blood samples were taken at 4-week intervals during the treatment and once at 24 weeks after the completion of treatment. The primary outcome measure was HBeAg seroconversion (defined by the loss of HBeAg and presence of anti-HBe in serum) 24 weeks after the completion of the 48-week treatment, which was defined as a treatment response. The secondary outcome measures were as follows: loss of HBeAg, loss of HBsAg, HBsAg seroconversion (defined as the loss of HBsAg and the presence of anti-HBs in serum), undetectable HBV DNA, and normalization of ALT levels 24 weeks after the completion of the 48-week treatment. Serum HBeAg, anti-HBe, and anti-HBs levels were measured by chemiluminescence immunoassay (Abbott Laboratories, Chicago, IL). Serum HBs levels were measured by using an ARCHITECT HBsQT assay kit (Abbott Laboratories), which has a lower limitation of detection of 0.05 IU/mL. Serum HBV DNA was quantified with the COBAS TaqMan HBV DNA test (Roche Diagnostics, Basel, Switzerland), which has a dynamic range of 2.1–9.0 log copies/mL. The conversion factor between HBV copies/mL and HBV IU/mL is considered to be 5.82 copies/IU. Genotyping of HBV was determined by the polymerase chain reaction (PCR)-Invader assay (12).

### Chemokine Secretion Assays

Serum samples for the evaluation of chemokines and cytokines could be obtained from 10 patients at 0, 12, 24, 36, and 48 weeks during the PEG-IFN treatment. We examined the serum levels of CXCL9 (MIG), CXCL10 (IP-10), CXCL11 (I-TAC), and CXCL13 (BCA1) using a Bio-Plex Pro Human Chemokine custom panel (Bio-Rad Laboratories, Hercules, CA). IL-21 was measured by ELISA (eBioscience).

### Vaccine Escape Mutant

Mother-to-child transmission due to immunoprophylaxis failure is the main source of pediatric chronic HBV infection in Japan. The S gene sequence was evaluated for the presence of vaccine escape mutants (VEMs). PCR was performed using HBV DNA derived from serum, and PCR products were analyzed by direct sequence (13). Moreover, G145R-specific real-time PCR was performed to identify G145R VEM as a minor population (13).

### Safety Analysis

Safety analysis included assessment of adverse events, growth parameters, hematologic/chemical measurements, and clinical/vital signs. Safety was assessed at baseline, every 4 weeks during treatment, and 6 months after the treatment. Z scores, the number of deviations from the mean height/weight for age, were calculated using cross-sectional growth charts (http://jspe.umin.jp/medical/chart_dl.html), which were provided by the Japanese Society for Pediatric Endocrinology.

### Statistics

Categorical variables were compared between groups using the Yates-corrected χ^2^ test or Fisher exact test. Noncategorical variables were compared between groups by the Mann–Whitney *U* test. All tests were 2-sided, and a *P* value of 0.05 or less was considered to indicate statistical significance. Statistical analyses were performed with StatMate IV for Windows (Advanced Technology for Medicine & Science, Tokyo), Microsoft Office Excel 2010, and Graph Pad Prism software (San Diego, CA).

## RESULTS

We offered high-dose PEG-IFN treatment to all patients and parents who were eligible for antiviral treatment. There was no selection of patients. Thirteen patients (gender, male/female = 4/9; age, 5–19 years; median, 9 years) with chronic HBV genotype C infection were enrolled in this study (Supplemental Table 1, Supplemental Digital Content, *http://links.lww.com/PG9/A0*).

### Efficacy of Treatment

**FIGURE 1.**
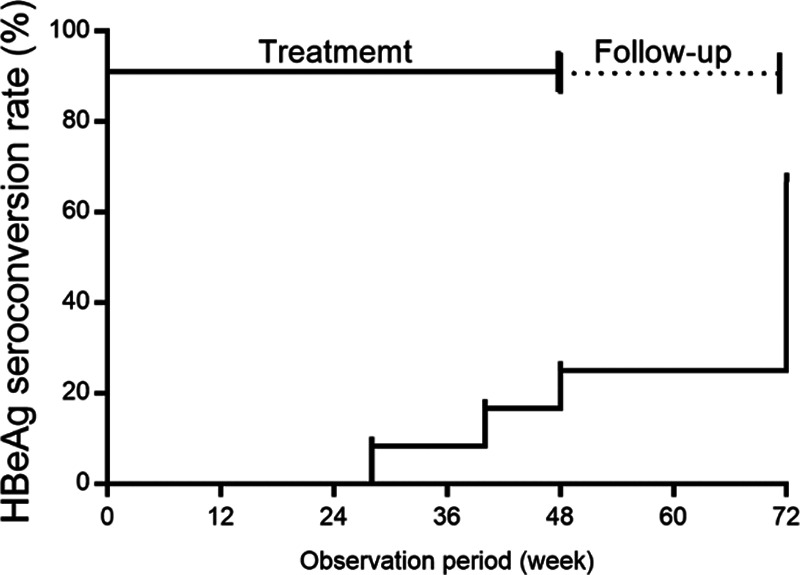
Rate of hepatitis B e antigen seroconversion in children treated with pegylated interferon during and after treatment.

**FIGURE 2.**
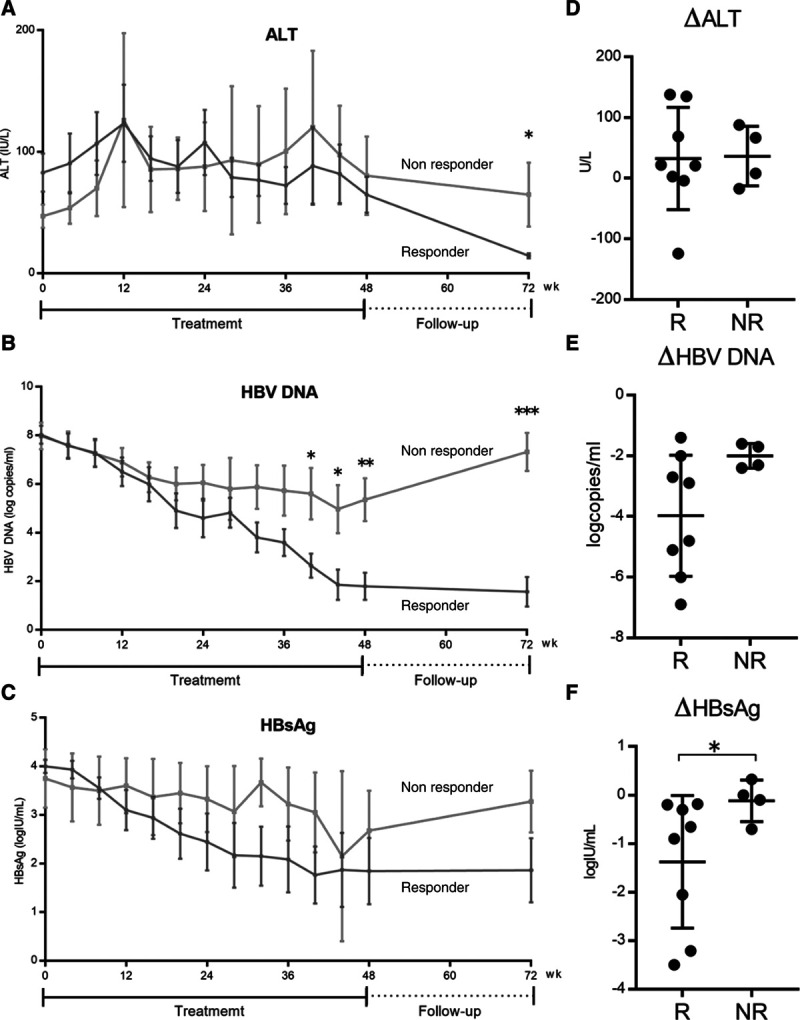
Dynamics of ALT (A), HBV DNA (B), and HBsAg (C) levels in children during 48 weeks of pegylated interferon treatment and during 24 weeks of follow-up (n = 12). The data at each time point are shown as means ± SE. The red and blue lines represent the responders (n = 8) and the nonresponders (n = 4), respectively. Comparison of the changes in ALT (D), HBV DNA (E), and HBsAg (F) at week 24 from the baseline (0 weeks) between the responders (R) (n = 8) and nonresponders (NR) (n = 4). *P < 0.05; **P < 0.01; ***P < 0.001 by Mann–Whitney test. ALT indicates alanine aminotransferase; HBsAg, hepatitis B surface antigen; HBV, hepatitis B virus.

Of the 13 patients, 1 (a 15-year-old girl) dropped out of PEG-IFN treatment due to adverse psychiatric effects (irritability and anxiety) 4 weeks after the initiation of treatment (Supplemental Fig. 1, Supplemental Digital Content, *http://links.lww.com/PG9/A0*). Therefore, 12 patients received PEG-IFN weekly for 48 weeks and were followed for 24 weeks after the treatment (Supplemental Table 2, Supplemental Digital Content, *http://links.lww.com/PG9/A0*). The treatment response results are shown in Table 1. Of the 12 patients, HBeAg seroconversion was observed in 5 (42%) at the end of treatment and 8 (67%) at the end of follow-up (Fig. 1, Table 1). ALT normalization had occurred in 4 (33%) at the end of treatment and 10 (83%) at the end of follow-up. Of the 10 patients with normalization of ALT at the end of follow-up, 8 were positive for anti-HBe. The peak ALT level was observed at week 12 of treatment in both responders and nonresponders (Fig. 2A). Serum HBV DNA was not detected in 3 patients (25%) at the end of treatment and in 4 (33%) at the end of follow-up (Table 1). All 4 patients with undetectable HBV DNA were positive for anti-HBe at the end of follow-up (Supplemental Table 2, Supplemental Digital Content, *http://links.lww.com/PG9/A0*).

**Table 1.**
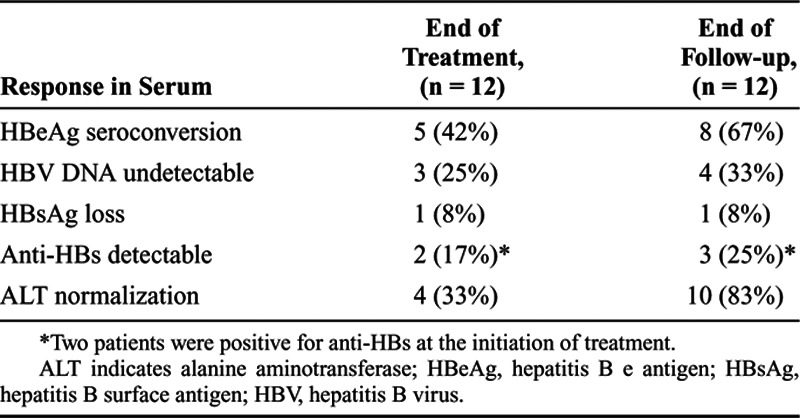
Response at the End of Treatment and Follow-up

The baseline characteristics of the 12 children receiving PEG-IFN are shown in Table 2. Although the number of subjects was small, the baseline characteristics showed no significant differences in age, gender, BSA, transmission route, HBV DNA level, HBsAg level, albumin level, or platelet count between the responders and nonresponders. Four children showed normal value of ALT at the initiation of PEG-IFN treatment. Of the 4 children, 2 achieved HBeAg seroconversion at the end of follow-up (Supplemental Table 2, Supplemental Digital Content, *http://links.lww.com/PG9/A0*). Although a significant difference (*P* < 0.05) was detected in total bilirubin level between the 2 groups at baseline, total bilirubin levels were normal in all children. All but 1 showed normal value (10 ng/ml) of alpha fetoprotein (AFP) at the baseline and at the end of follow-up. One patient showed an increase of AFP value (67.5 ng/mL) before PEG-IFN therapy. Abnormal finding of liver was not detected by abdominal ultrasound. After the initiation of PEG-IFN, the level of AFP was decreased to normal range and remained normal value at the end of follow-up.

**Table 2.**
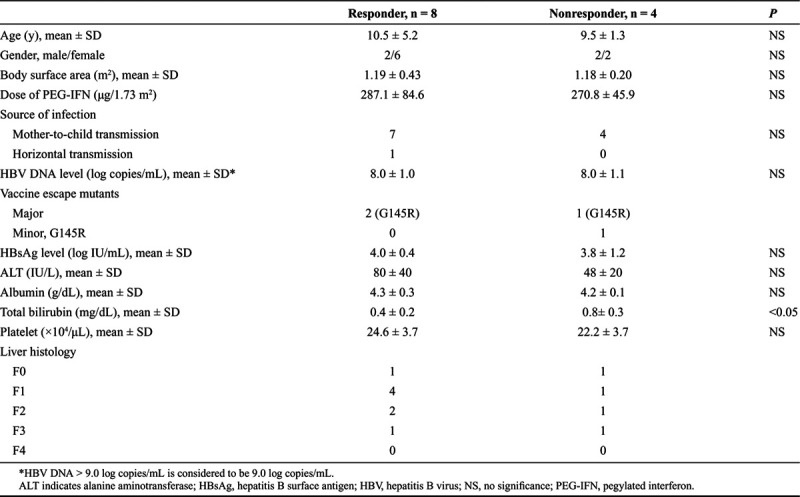
Baseline Characteristics of the Patients Receiving PEG-IFN According to Treatment Response

The kinetics of the serum HBV DNA level during the treatment and follow-up are shown in Figure 2B. Both the responders and the nonresponders showed a gradual decline in the HBV DNA level. However, a marked increase in the HBV DNA level was observed in nonresponders after the end of treatment. Only 1 patient became negative for HBsAg at the end of treatment and during follow-up. Although 3 patients (21%) were positive for anti-HBs at the end of treatment and during follow-up, 2 patients (responder, n = 1; nonresponder, n = 1) were already positive for anti-HBs before the initiation of treatment (Supplemental Table 2, Supplemental Digital Content, *http://links.lww.com/PG9/A0*). Both patients had G145R VEM as a major strain. The kinetics of the serum HBsAg level during the treatment and follow-up are shown in Figure 2C. In the nonresponders, the levels of HBV DNA and HBsAg increased after 48 weeks of treatment (Fig. 2B, C).

To investigate on-treatment biomarkers to predict the success of PEG-IFN therapy in children and adolescents, we compared ALT, HBV DNA, and HBsAg levels at weeks 0, 4, 8, 12, 16, 20, and 24 between the responders and nonresponders. There were no significant differences in the levels of ALT, HBV DNA, or HBsAg between responders and nonresponders at any of these time points (Fig. 2A–C). We also evaluated the change in these 3 parameters from the start of treatment to each time point. The changes from the baseline to week 24 were as follows: ALT: responders, 36.5 ± 49.2 IU/L; nonresponders, 32.2 ± 84.4 IU/L; HBV DNA: responders, –4.0 ± 2.0 log copies/mL; nonresponders, –2.0 ± 0.41 log copies/mL; HBsAg: responders, –1.38 ± 1.37 log IU/mL; nonresponders, –0.12 ± 0.42 log IU/mL. There were no significant differences in the changes of the ALT and HBV DNA levels from the baseline to week 24 between the 2 groups (ALT, *P* = 0.934; HBV DNA, *P* = 0.067; Fig. 2D, E). Compared with nonresponders, however, the responders showed a significant decrease in HBsAg from the baseline to week 24 (*P* = 0.049; Fig. 2F).

### Adverse Events

One patient dropped, but the remaining 12 patients had no serious adverse events. Fever, injection site rejection, headache, general fatigue, nasal bleeding, and arthralgia were observed in 30% or more of the children (Supplemental Table 3, Supplemental Digital Content, *http://links.lww.com/PG9/A0*). The frequencies of the adverse events were comparable to those of dose-adjusted PEG-IFN therapy (5,14). None of the patients showed ALT flare (>5 × ULN) during and after treatment. During PEG-IFN treatment, the lowest levels of a leukocyte count, granulocyte count, and platelet count were 2500/μL, 11,750/μL, and 104,100/μL in the patients. Therefore, there was no patient who needed PEG-IFN dose reduction due to cytopenia. The *z* scores of height-for-age and weight-for-age in 10 children (age, 5–11 years; median, 9 years) treated with PEG-IFN during and after the treatment are shown in Supplemental Fig. 2, Supplemental Digital Content, *http://links.lww.com/PG9/A0*. The *z* scores of height-for-age and weight-for-age showed a decline during the treatment. However, catch-up growth occurred in the patients’ height and weight by the end of the follow-up period.

### Cytokine and Chemokine Response

Serum CXCL9, CXCL10, CXCXL11, CXCL13, and IL-21 were longitudinally compared between the responders (n = 7; cases 1, 2, 3, 4, 6, 7, 8) and nonresponders (n = 3; cases 10, 11, 12) through the 48 weeks of treatment (Supplemental Table 2, Supplemental Digital Content, *http://links.lww.com/PG9/A0*). The levels of CXCL9, CXCL10, CXCL11, and CXCL13 in the responders were higher but not significantly higher than those in the nonresponders during the first 24 weeks of treatment (Supplemental Fig. 3, Supplemental Digital Content, *http://links.lww.com/PG9/A0*). IL-21 was detected in 4 of the 7 responders and all 3 nonresponders, and no significant differences were observed between the groups. In case 1 (a 9-year-old boy), HBeAg and HBsAg became negative, and anti-HBs became positive at the end of the 48-week treatment. CXCL9, CXCL10, CXCL11, and CXCL13 were increased after 24 weeks. IL-21 increased at weeks 12 and 48. Subsequently, HBsAg and HBeAg became negative at weeks 40 and 48, respectively (Supplemental Fig. 4, Supplemental Digital Content, *http://links.lww.com/PG9/A0*). In contrast, in case 12 (an 8-year-old girl) who failed to attain HBeAg seroconversion or to show a reduced HBsAg level in the course of PEG-IFN therapy, such dynamic changes of CXCL9, CXCL10, CXCL11, and CXCL13 were not observed. These cases suggest that the increase in the serum levels of CXCL9, CXCL10, CXCL11, and CXCL13 were associated with attaining an HBsAg loss in children with chronic hepatitis B.

## DISCUSSION

In this study, a high dose of PEG-IFN therapy was effective and well tolerated in HBeAg-positive children and adolescents with chronic HBV genotype C infection. This study showed that HBeAg seroconversion and HBsAg loss occurred in 67% and 8% of patients with chronic HBV infection at 24 weeks after treatment, respectively. A randomized controlled pediatric trial of PEG-IFN-α-2a 48-week treatment for HBeAg-positive children with chronic HBV infection, in which HBV genotypes A to E were included, showed that the rates of HBeAg and HBsAg loss were 25.7% and 8.9% at 24 weeks posttreatment, respectively (5). In children with genotype C, the rate of HBeAg seroconversion was 38.2% (5). The rate of HBeAg seroconversion in our present study was 2 times higher than that in the pediatric trial. In adults infected with genotype C, the rates of HBeAg loss were 31% at 24 weeks of a PEG-IFN-α-2a 48-week treatment (7) and 28% at 24 weeks of a PEG-IFN-α-2b 52-week treatment (8). The rate of HBeAg loss in this study was thus twice as high as that in adult studies.

In contrast, the rate of HBeAg seroconversion in this study was consistent with recent pediatric studies from China (14–16). The 3 pediatric Chinese studies showed that the rate of HBeAg loss ranged from 66.7% to 72.1% in HBeAg-positive children who underwent a 48- to 52-week PEG-IFN treatment. Moreover, these studies showed that HBsAg loss occurred in approximately 50% of children treated with PEG-IFN (14,16). Although the children were treated with a high dose of PEG-IFN (180–311 μg/1.73 m^2^) in 1 of the 3 Chinese studies, the distribution of HBV genotypes was not described (16). Although it is difficult to explain the discrepancy in the rates of HBsAg between this study and the Chinese studies, the difference in the distribution of HBV genotypes might have contributed to the difference in the rates of HBsAg loss. Of the 8 patients who achieved HBeAg seroconversion, 4 remained positive for serum HBV DNA at the end of follow-up in this study. This finding suggests that the ideal endpoint of treatment should be sustained HBsAg clearance. Longer terms of posttreatment monitoring are necessary.

High pretreatment ALT levels, low pretreatment HBV DNA levels, sex, age, and HBV genotype A or B are considered predictors of a good response to PEG-IFN treatment for HBeAg-positive chronic hepatitis B patients (8,17–20). This present study showed a significant difference in the pretreatment levels of total bilirubin between responders and nonresponders. However, the pretreatment levels of total bilirubin were normal in all children. Therefore, it seems that total bilirubin is not a predictor of PEG-IFN treatment. Half (6/12) of the patients showed no or mild hepatic fibrosis (F0 and F1). Liver histology might contribute to the high rate of HBeAg seroconversion.

In case 1, who achieved HBsAg loss, increases in CXCL9, CXCL10, CXCL11, and CXCL13 were observed with ALT elevation and subsequent decreases in HBsAg and HBV DNA (Supplemental Fig. 4, Supplemental Digital Content, *http://links.lww.com/PG9/A0*). In contrast, in the nonresponder (case 12), no elevation of such chemokines was observed during the treatment, and no reduction in HBsAg or HBV DNA was observed (Supplemental Fig. 4, Supplemental Digital Content, *http://links.lww.com/PG9/A0*). Both cases showed normal value of ALT at the initiation of treatment. Thus, ALT value at the initiation of treatment might not be able to predict PEG-IFN-α-induced activation of innate and adaptive immunity. Further studies are necessary to determine whether pretreatment ALT value is associated with activation of PEG-IFN-α-induced immunity.

At enrollment, VEMs were detected in 5 (38%) of 13 children. All of the 5 children were infected with HBV due to immunoprophylaxis failure. It is unknown whether the presence of VEMs is disadvantageous to achieving a response to PEG-IFN. One child who dropped out of the PEG-IFN treatment was infected with VEMs. Of the remaining 4 patients, 2 (50%) achieved HBeAg seroconversion. Therefore, children infected with VEMs should be treated if they are indicated for antiviral treatment. In other words, it might be unnecessary to evaluate whether VEMs are present in patients at the initiation of PEG-IFN therapy. There was no severe adverse event in this study. It was confirmed that high-dose PEG-IFN therapy was safe and tolerable in HBeAg-positive children with chronic HBV genotype C infection just as it was in children with HCV infection (9). However, pros and cons of antiviral agents should be discussed with the family before making a decision. When patient and family would prefer long-term oral administration to PEG-IFN, age-dependent approved nucleos(t)ide analogues with high genetic barrier to resistance can be considered the first-line treatment (21).

In conclusions, high-dose PEG-IFN therapy was shown to benefit HBeAg-positive children with chronic HBV genotype C infection. The decrease of HBsAg levels from the baseline to week 24 might be a predictor of HBeAg seroconversion.

## ACKNOWLEDGMENTS

H.K., A.I., S.Y., and T.K. contributed to the design of the study and drafted the article. A.I., S.Y., S.U., T.T., and T.F. participated in the data collection and critically revised the article. All of the authors concurred with the submission and take responsibility for the article.

## Supplementary Material


