# Supplementary material for: High Dose of Pegylated Interferon for the Treatment of Chronic Hepatitis B in Children Infected With Genotype C
Source: JPGN Rep. 2020 Aug 19;1(2):e005. doi: 10.1097/PG9.0000000000000005 (PMC10191545; doi:10.1097/PG9.0000000000000005)

Supplemental Table 1.

Baseline characteristics of the patients receiving PEG-IFN

	n=13
Age, years, mean±SD	10.5±4.3
median	9 (5-19)
Gender, Male/Female	4/9
Body surface area, m^2, mean±SD	1.21±0.36
median	1.09 (0.76-2.01)
Transmission mode of HBV infection	
Mother-to-child transmission	12
Horizontal transmission	1
HBeAg positive	13
HBV genotype	
C	13
HBV DNA level, log copies/mL, mean±SD†	7.9±1.0
median	8.3 (6.2->9)
Vaccine escape mutants	
Major	4 (3;G145R, 1;G145K)
Minor, G145R	2
HBsAg level, log IU/mL, mean±SD	3.7±0.5
median	4.0 (3.2-4.7)
ALT, IU/L, mean±SD	70 ± 36
Albumin, g/dL, mean±SD	4.3 ± 0.3
Total bilirubin, mg/dL, mean±SD	0.6 ± 0.3
Platelet, $\times 10^4$ /μL, mean±SD	23.5 ± 3.7
Liver histology	
F0	3
F1	5
F2	3
F3	2
F4	0

†HBV DNA>9.0 log copies/mL is considered to be 9.0 log copies/mL.

Supplemental Table 2. Characteristics of 12 patients who received PEG-IFN therapy for 48weeks

Case	HBV genotype	Gender	Age 0w, year	Vaccine escape mutant	HBeAg 0w	HBsAg 0w,log IU/mL	anti-HBs 0w	HBV DNA 0w, log copies/ml	ALT 0w, IU/L	⊿HBsAg (24w-0w)	HBeAg seroconversion 72w	HBsAg loss 72w	anti-HBs 72w	HBVDNA 72w	ALT normalization 72w
1	C	F	9	-	+	4.3	-	6.9	27	-3.2	+	+	+	-	+
2	C	M	9	-	+	4.0	-	8.3	67	-3.5	+	-	-	-	+
3	C	F	10	G145R major	+	3.6	+	7.5	86	-2.1	+	-	+	-	+
4	C	F	5	G145R major	+	4.4	-	9	141	-0.9	+	-	-	-	+
5	C	F	9	G145R major	+	2.0	+	6.4	26	-0.7	-	-	+	+	+
6	C	F	6	-	+	4.5	-	9	87	-0.7	+	-	-	+	+
7	C	F	8	-	+	3.7	-	8.4	64	-0.3	+	-	-	+	+
8	C	M	18	-	+	3.5	-	8.9	165	-0.2	+	-	-	+	+
9	C	F	19	-	+	4.0	-	6.2	26	-0.2	+	-	-	+	+
10	C	M	11	G145R minor	+	4.0	-	8.3	63	-0.1	-	-	-	+	+
11	C	M	10	-	+	4.3	-	9	64	0.0	-	-	-	+	-
12	C	F	8	-	+	3.7	-	8.2	35	0.3	-	-	-	+	-

Supplemental Table 3.

Adverse effects in patients treated with PEG-IFN

	n=13	(%)
Fever	9	(69)
Injection site reaction	6	(46)
General fatigue	5	(38)
Headache	4	(31)
Nasal bleeding	4	(31)
Arthralgia	4	(31)
Dry skin	3	(23)
Diarrhea	3	(23)
Alopecia	3	(23)
Itching	3	(23)
Loss of appetite	2	(15)
Irritability and anxiety	1	(8)
Depression	1	(8)
Dizziness	1	(8)

Supplemental Fig. 1. Flowchart of study

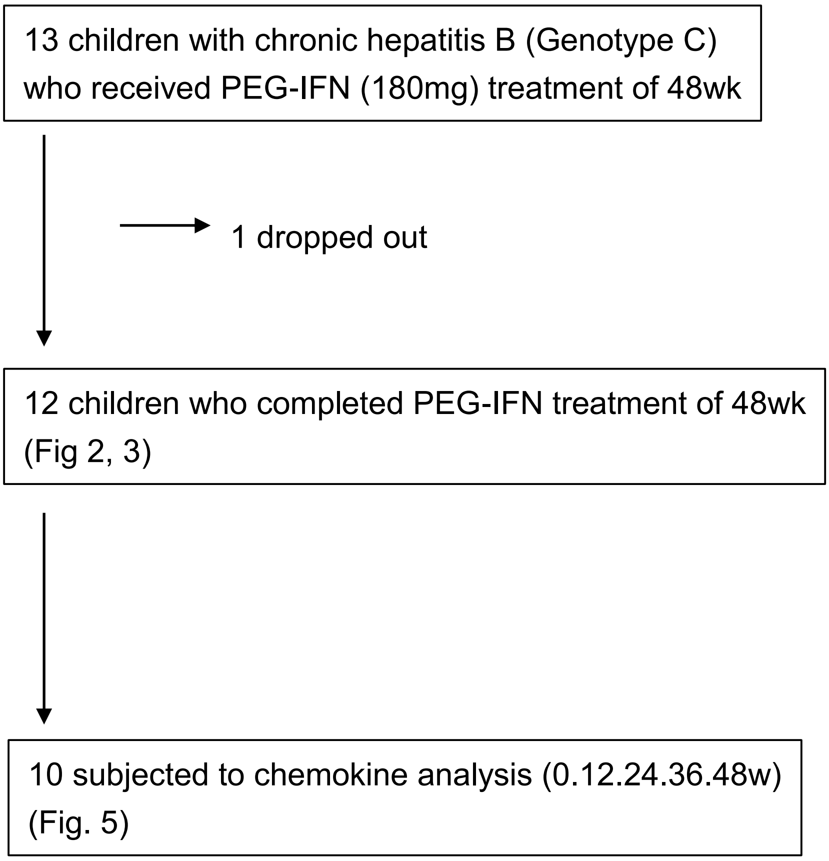

Supplemental Fig. 2. Change in mean (A) z-score of height-for-age and (B) weight-for-age in children treated with pegylated interferon during and after treatment. The data at each time point are shown as means±SE.

(A)

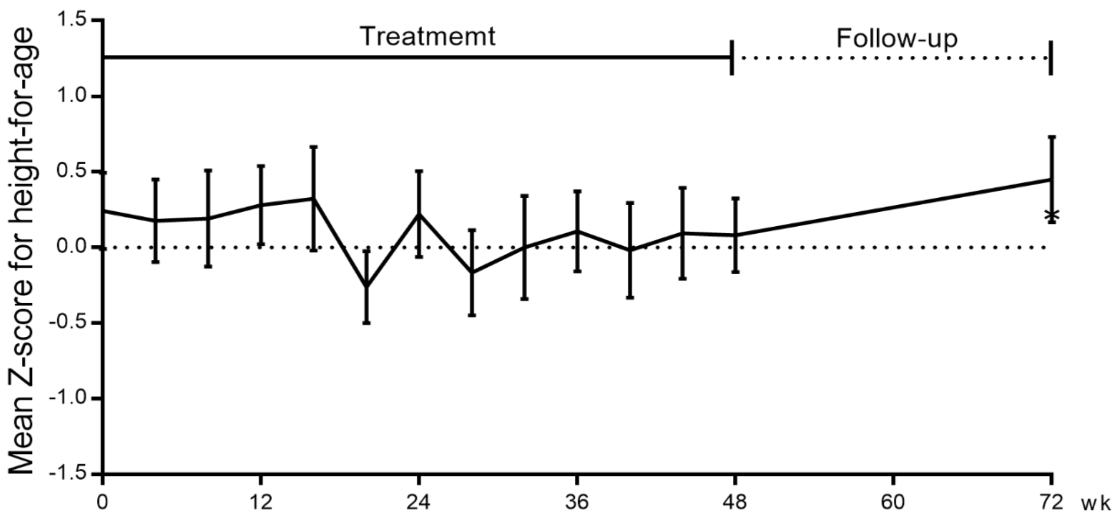

(B)

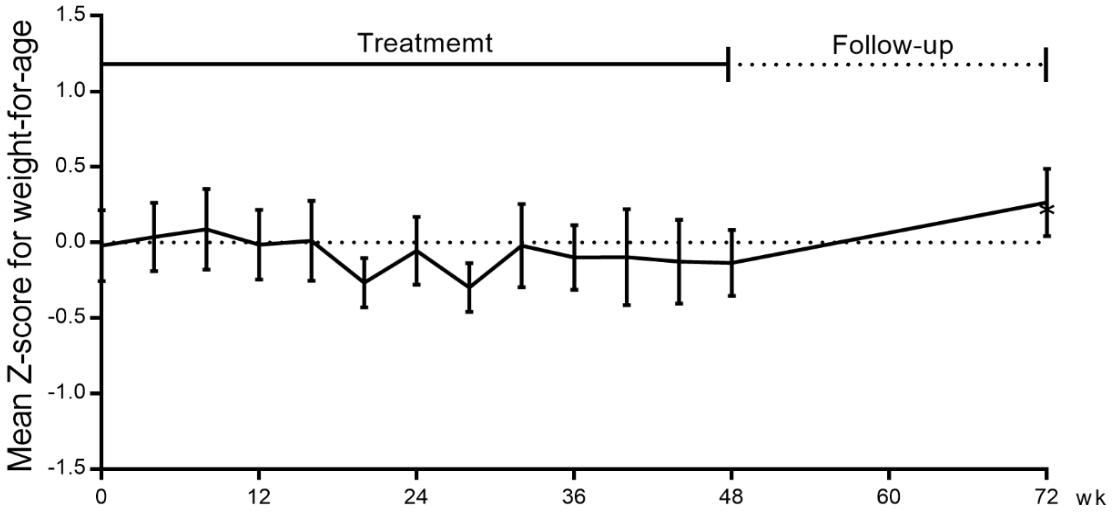

Supplemental Fig. 3. Dynamics of HBsAg, HBV DNA, ALT, CXCL9, CXCL10, CXCL11, CXCL13, and IL-21 among children with chronic hepatitis B treated with pegylated interferon (n=10; 7 responders and 3 non-responders). The X-axis indicates the weeks after the start of PEG-IFN treatment. Day 0 represents the initiation of PEG-IFN treatment. The Y-axes indicate the levels of serum CXCL9, CXCL10, CXCL11, CXCL13, and IL-21. The red and blue solid lines indicate the average±SE values in the responders and non-responders, respectively. *p<0.05 by the Mann-Whitney test.

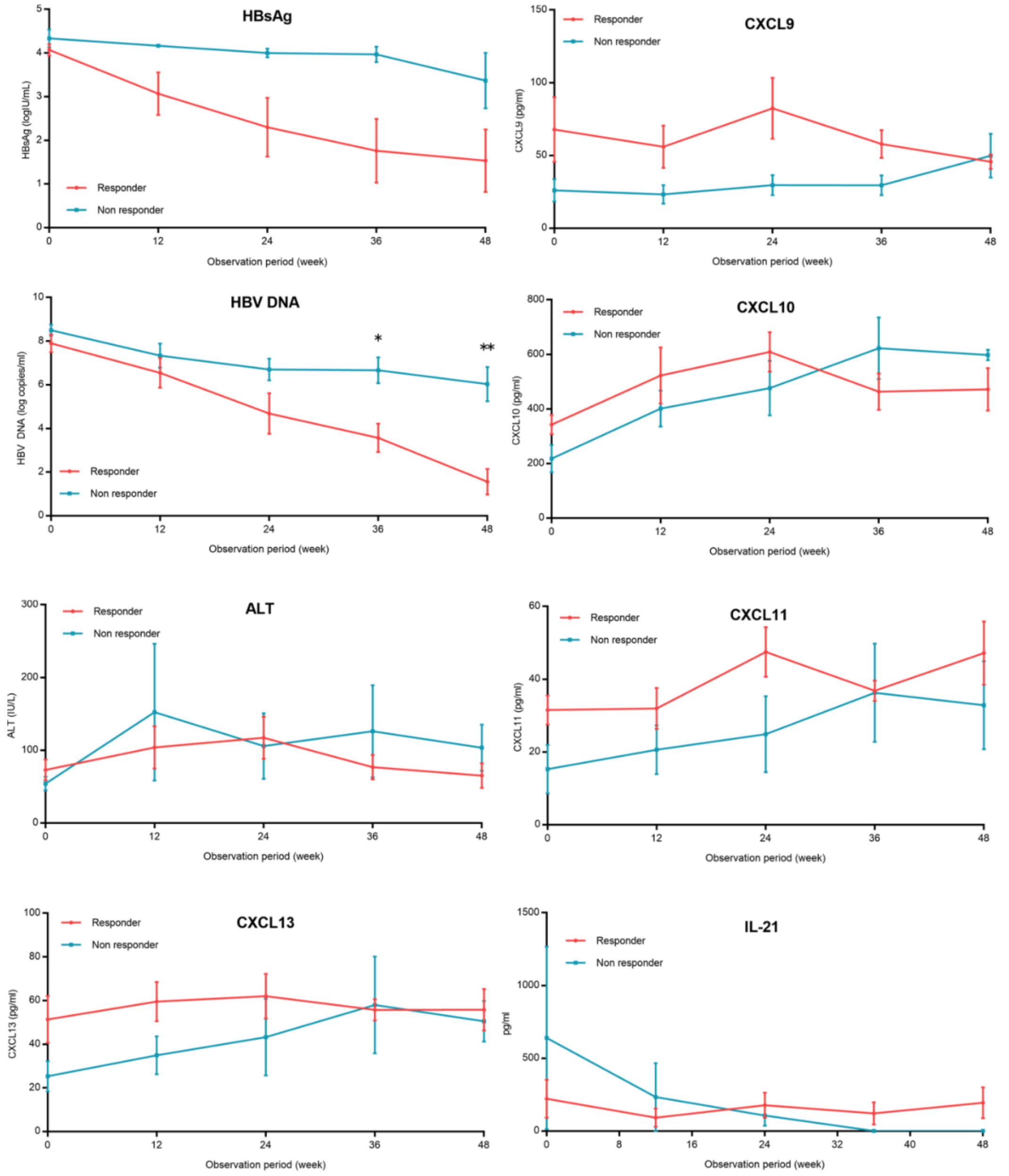

Supplemental Fig.4. Time course of HBV markers, ALT, and chemokines in the responders who achieved HBsAg loss. Case 1 (9 years old, female) was positive for HBeAg, HBsAg (4.3 log IU/mL), and HBV DNA (6.9 log copies/mL) at the start of PEG-IFN therapy. At the end of treatment (48 weeks), he became positive for anti-HBe and anti-HBs. Case 12 (8 years old, female) was positive for HBeAg, HBsAg (3.7 log IU/mL), and HBV DNA (8.2 log copies/mL) at the start of treatment. At the end of treatment (48 weeks), HBsAg and HBV DNA remained positive. The X-axis indicates the weeks after the start of the PEG-IFN treatment. Day 0 represents the initiation of PEG-IFN treatment. The Y-axes indicate the levels of serum HBsAg, HBV DNA, ALT, CXCL9, CXCL10, CXCL11, and CXCL13.

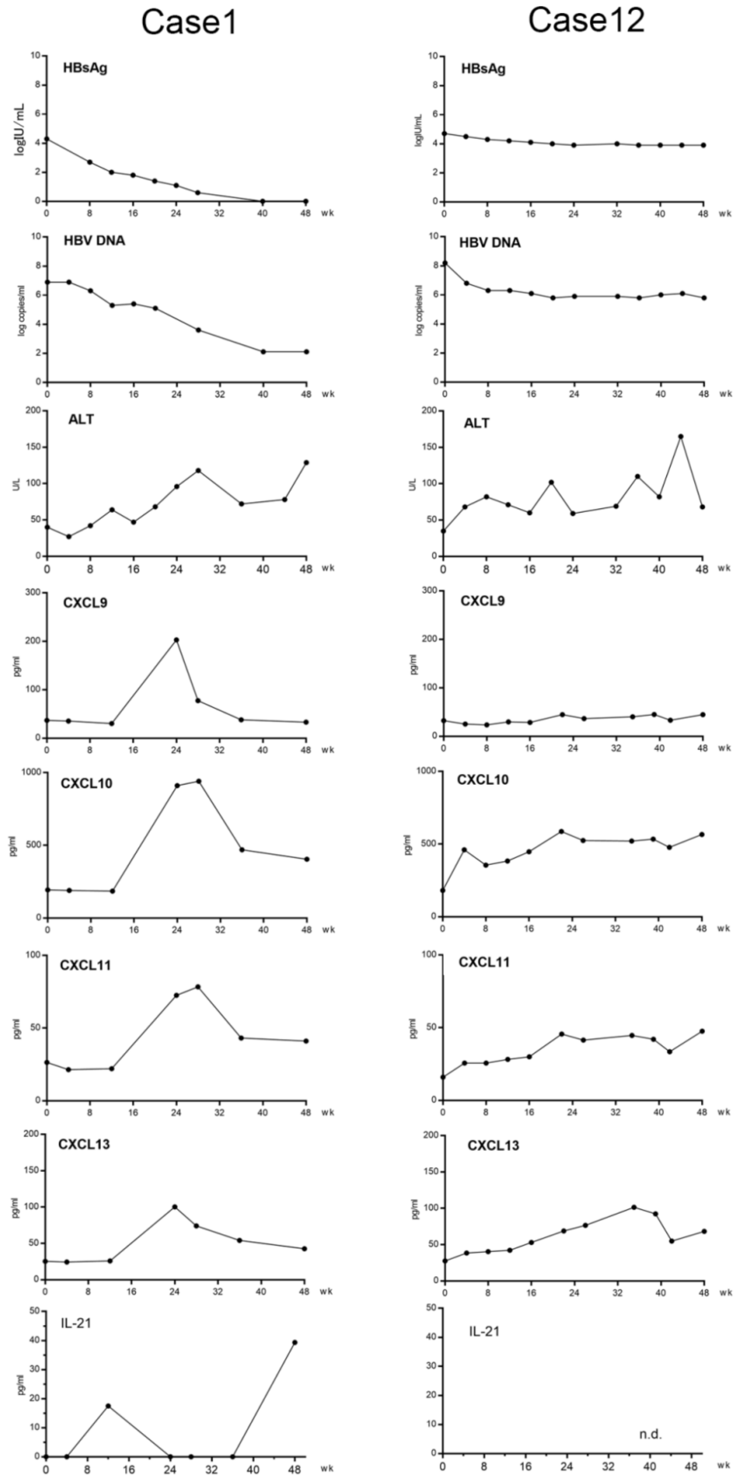

Supplement: Supplementary file 1 [file pg9-1-e005-s001.pdf]
